# Large Follicular Odontogenic Keratocyst affecting Maxillary Sinus mimicking Dentigerous Cyst in an 8-year-old Boy: A Case Report and Review

**DOI:** 10.5005/jp-journals-10005-1537

**Published:** 2018-08-01

**Authors:** Madhusudhan R Madhireddy, A Jacod Prakash, Vijayalakshmi Mahanthi, K Venkata Chalapathi

**Affiliations:** 1 Reader, Department of Oral Pathology and Microbiology, Army College of Dental Sciences, Secunderabad, Telangana, India; 2 Reader, Department of Oral Pathology and Microbiology, KLR’s Lenora Institute of Dental Sciences, Rajahmundry, Andhra Pradesh, India; 3 Senior Lecturer, Department of Oral Pathology and Microbiology, KLR’s Lenora Institute of Dental Sciences, Rajahmundry, Andhra Pradesh, India; 4 Reader, Department of Oral Pathology and Microbiology, Care Dental College, Guntur, Andhra Pradesh, India

**Keywords:** Dentigerous cyst, Follicular odontogenic kerato-cyst, Keratocystic odontogenic tumor.

## Abstract

Cyst with relatively high recurrence and having nature to invade the underlying tissue is “odontogenic keratocyst (OKC).” Radiographically, OKC can appear as different varieties, such as follicular, replacemental, extraneous, envelopmental, and collateral. Each radiographic variety of OKC varies in biological behavior, prognosis, recurrence, and therapeutic approaches.

Many studies done till date have not established any relationship between markers of proliferation and aggressiveness in regard to radiographic varieties of OKC. The present article reports a case of follicular OKC in an 8-year-old boy which was concluded as a dentigerous cyst by radiographic features.

**How to cite this article:** Madhireddy MR, Prakash AJ, Mahanthi V, Chalapathi KV. Large Follicular Odontogenic Keratocyst affecting Maxillary Sinus mimicking Dentigerous Cyst in an 8-year-old Boy: A Case Report and Review. Int J Clin Pediatr Dent 2018;11(4):349-351.

## INTRODUCTION

Keratocystic odontogenic tumor (KCOT), formerly known as OKC, was first named by Philipsen.^1^

According to the World Health Organization (WHO) updated classification of Head and Neck Tumors, published in 2005, the use of term KCOT is appropriate, as it reflects its neoplastic nature and it is defined as “a benign uni- or multicystic, intraosseous tumor of odontogenic origin, with a characteristic lining of parakeratinized stratified squamous epithelium and has the potential for aggressive, infiltrative behaviour.”

Other synonyms include odontogenic keratocystoma and primordial cyst.^2^ It is a cystic jaw lesion which is locally aggressive and having high growth potential and recurrence rate.^3^ It demonstrates a bimodal age distribution, first peak in the 2nd and 3rd decades and second peak in the 5th decade or later and are more common in males.

The mandible is the most common site.^1^ Reported recurrences range from 0 to 100%.^4^ Its pathognomonic microscopic features, potential aggressiveness, and its high association with nevoid basal cell carcinoma syndrome (NBCS) or Gorlin-Goltz syndrome makes this cyst unique among the odontogenic cysts.^5^

Radiographical presentation of OKCs is variable, showing extensive involvement with little or no bony expansion. About half of all OKCs occur at the angle and ramus of the mandible and present as unilocular or mul-tilocular radiolucencies, but other odontogenic lesions may show similar radiological findings.

Follicular keratocyst is a variant of OKC in which cyst surrounds the crown of an unerupted tooth and is attached to the neck of the tooth. Follicular OKC accounts for about 25 to 40% of all OKCs.^6^ Thus, a few clinical and radiographic features are specific for OKCs. The final diagnosis depends on histological examination.^5^

## CASE REPORT

**Fig. 1:**
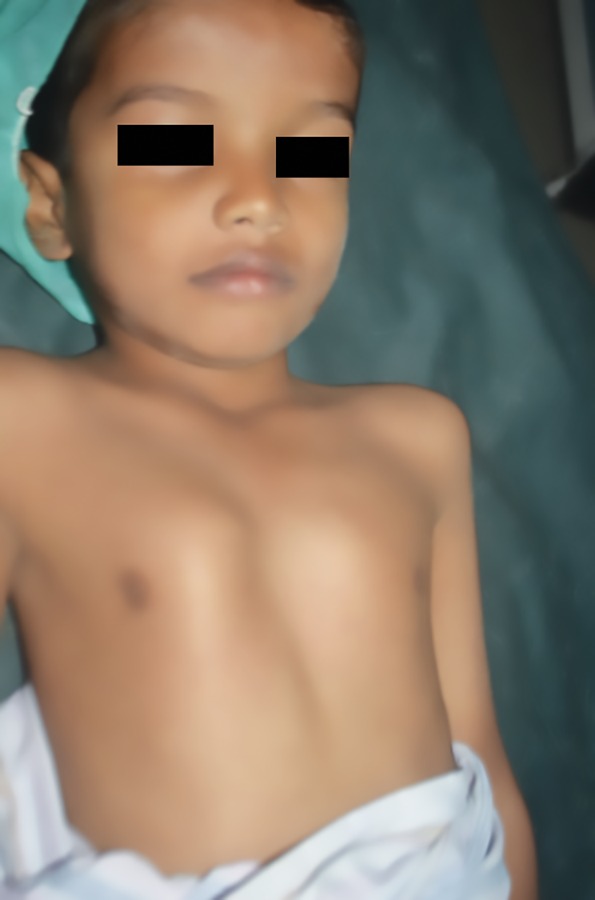
General examination reveals pectus carinatum (pigeon breast) and gross facial asymmetry

An 8-year-old male patient reported to a dental clinic with the chief complaint of swelling in the upper right front tooth region since 1 year. Extraoral examination showed slight swelling was present on the right side of face, causing elevation of right ala of nose (Fig. 1). General examination, gross facial asymmetry, and other findings were noncontributory; there was no history of trauma.

**Fig. 2:**
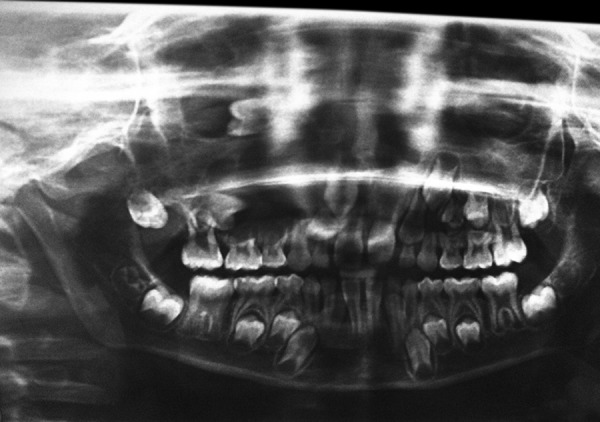
Panoramic radiograph showing cystic lesion in the right maxilla

The panoramic radiograph of the patient showed mixed dentition with tooth buds at different developmental stages. A large radiolucent lesion of 2 × 2 cm in size was found in the right side of face area in relation to periapical region of 54 and 55. Tooth bud of 14 was being pushed close to the region of floor of the orbit and tooth bud of 15 being pushed posteriorly (Fig. 2).

**Fig. 3:**
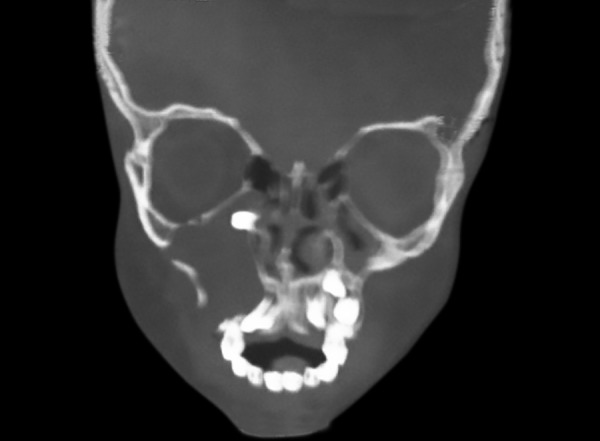
Computed tomography coronal view showing cystic occupying maxillary sinus and tooth bud displaced close to the orbital floor

**Fig. 4:**
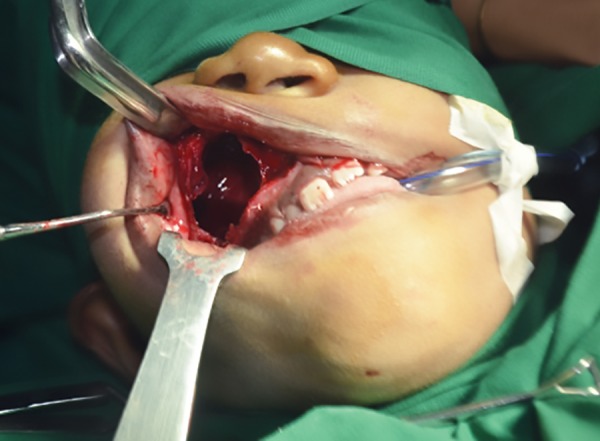
Surgical excision under general anesthesia

**Fig. 5:**
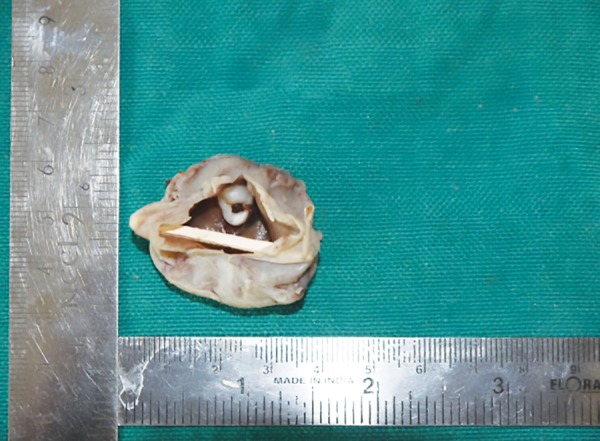
Surgically excised cyst attached to the tooth

The borders of the lesion are ill defined. Coronal computed tomography showed a radiolucent lesion in relation to right maxillary sinus area with tooth bud being displaced close to the region below the floor of the orbit (Fig. 3). Considering the clinical and radiologic presentations, a provisional diagnosis of dentigerous cyst was determined.

The patient underwent surgery under general anesthesia (Fig. 4). A mucoperiosteal flap was opened; the entire cyst lining was enucleated along with the fibrous capsule (Fig. 5) and was sent for histopathological examination. Histopathological examination revealed odontogenic epithelial lining of 6 to 9 cell thickness, which exhibited a wavy appearance.

**Fig. 6:**
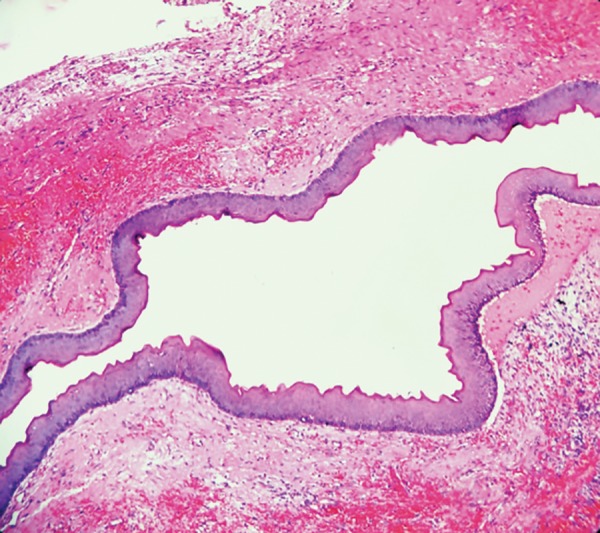
Hematoxylin and eosin-stained section showing odontogenic epithelium characteristic of OKC

The basal epithelial layer was composed of palisaded columnar cells. There was also presence of small satellite cystic islands of odontogenic epithelium seen in the fibrous connective tissue. Histopathological features were suggestive of KCOT (Fig. 6).

## DISCUSSION

The OKC was named by Phillipsen in the year 1956. However, it was renamed as keratocystic odontogenic tumor by the WHO in the year 2005. They are noninflammatory developmental tumors and odontogenic in origin.^7^ It is very aggressive in nature and known for its high recurrence rate and is associated with NCBS. The NCBS syndrome, first delineated by Gorlin and Goltz (1960), is characterized by basal cell carcinoma, OKC, palmar, plantar pits, and ectopic calcification of falx cerebri; 75% of patients affected by NBCS show multiple bilateral keratocysts.

Mainly located in premolar area, it may displace teeth with consequent malocclusion.^8^ It is more common in females and occurs over a wide range of age. In our case, the patient is in his first decade. The tumor most commonly occurs in molar region; however, in the present case, there was maxillary involvement with displacement of tooth bud superiorly into the maxillary antrum. Clinical features include pain, soft tissue swelling expansion of bone with gross facial asymmetry.

The PTCH1 mutations and dysregulation of sonic hedgehog pathway have a definite part in the development of OKCs in both isolated and syndromic cases.^9^ Radiographically, most KCOTs are unilocular, presenting a well-defined radiolucency with peripheral rim with scalloped borders. In the present case, the radiographic evaluation revealed an aggressive osteolytic lesion which has not only involved the maxillary sinus but also caused displacement of tooth bud of 44 into the sinus up to the extent of floor of the orbital cavity.

Histopathologically, it is composed of a palisade, polarized layer of cuboidal cells which are often hyper-chromatic and described as picket fence or tombstone appearance with folding of epithelium into capsule by active proliferation.^10^ Expression of high epidermal growth factor receptor in KCOTs has supported its intrinsic growth potential, which is not present in other odontogenic cysts.^11^ Treatment of KCOTs includes many surgical modalities, such as enucleation with primary closure, enucleation with open packing, marsupialization, enucleation with use of Carnoy’s solution, or cryotherapy, with a marginal or radical section.

Electro cauterization is used as a good adjunct for enucleation of KCOT lesions.^12^ Even though KCOTs are most aggressive and recurrent form of tumors, there are few cases where KCOTs have been treated by enucleation. The case discussed here also has been treated with the enucleation procedure. However, a long-term follow-up is required to establish nonrecurrence of the tumor.

## CONCLUSION

Clinical and radiographic features of some cases can mimic each other and cause diagnostic dilemmas in arriving at a final diagnoses. Histopathological examination is a gold standard for the final diagnoses.
